# Proton versus photon therapy for high-risk prostate cancer with dose escalation of dominant intraprostatic lesions: a preliminary planning study

**DOI:** 10.3389/fonc.2023.1241711

**Published:** 2023-11-08

**Authors:** Ashley Li Kuan Ong, Kellie Knight, Vanessa Panettieri, Mathew Dimmock, Jeffrey Kit Loong Tuan, Hong Qi Tan, Caroline Wright

**Affiliations:** ^1^ Division of Radiation Oncology, National Cancer Centre Singapore, Singapore, Singapore; ^2^ Department of Medical Imaging and Radiation Sciences, Monash University, Clayton, VIC, Australia; ^3^ Department of Physical Sciences, Peter MacCallum Cancer Centre, Melbourne, VIC, Australia; ^4^ Sir Peter MacCallum Department of Oncology, The University of Melbourne, Victoria, VIC, Australia; ^5^ Central Clinical School, Monash University, Melbourne, VIC, Australia; ^6^ School of Allied Health Professions, Keele University, Staffordshire, United Kingdom

**Keywords:** proton therapy, intraprostatic lesions, high-risk prostate cancer, biological modeling, magnetic resonance imaging

## Abstract

**Background and purpose:**

This study aimed to investigate the feasibility of safe-dose escalation to dominant intraprostatic lesions (DILs) and assess the clinical impact using dose-volume (DV) and biological metrics in photon and proton therapy. Biological parameters defined as late grade ≥ 2 gastrointestinal (GI) and genitourinary (GU) derived from planned (*D*_P_) and accumulated dose (*D*_A_) were utilized.

**Materials and methods:**

In total, 10 patients with high-risk prostate cancer with multiparametric MRI-defined DILs were investigated. Each patient had two plans with a focal boost to the DILs using intensity-modulated proton therapy (IMPT) and volumetric-modulated arc therapy (VMAT). Plans were optimized to obtain DIL coverage while respecting the mandatory organ-at-risk constraints. For the planning evaluation, DV metrics, tumor control probability (TCP) for the DILs and whole prostate excluding the DILs (prostate-DILs), and normal tissue complication probability (NTCP) for the rectum and bladder were calculated. Wilcoxon signed-rank test was used for analyzing TCP and NTCP data.

**Results:**

IMPT achieved a higher *D*mean for the DILs compared to VMAT (IMPT: 68.1 GyRBE vs. VMAT: 66.6 Gy, *p* < 0.05). Intermediate–high rectal and bladder doses were lower for IMPT (*p* < 0.05), while the high-dose region (V60 Gy) remained comparable. IMPT-TCP for prostate-DIL were higher compared to VMAT (IMPT: 86%; α/β = 3, 94.3%; α/β = 1.5 vs. VMAT: 84.7%; α/β = 3, 93.9%; α/β = 1.5, *p* < 0.05). Likewise, IMPT obtained a moderately higher DIL TCP (IMPT: 97%; α/β = 3, 99.3%; α/β = 1.5 vs. VMAT: 95.9%; α/β = 3, 98.9%; α/β = 1.5, *p* < 0.05). Rectal *D*_A_-NTCP displayed the highest GI toxicity risk at 5.6%, and IMPT has a lower GI toxicity risk compared to VMAT-predicted Quantec-NTCP (*p* < 0.05). Bladder *D*_P_-NTCP projected a higher GU toxicity than *D*_A_-NTCP, with VMAT having the highest risk (*p* < 0.05).

**Conclusion:**

Dose escalation using IMPT is able to achieve a high TCP for the DILs, with the lowest rectal and bladder DV doses at the intermediate–high-dose range. The reduction in physical dose was translated into a lower NTCP (*p* < 0.05) for the bladder, although rectal toxicity remained equivalent.

## Highlights

• Intensity modulated proton therapy (IMPT) obtained a higher average Dmean (68.1 Gy) compared to volumetric-modulated arc therapy (VMAT) (66.6 Gy)

• IMPT attained a higher dominant intraprostatic lesions (DILs) tumor control probability (99.3%) compared to VMAT (98.9 Gy)

• Rectal and bladder for IMPT received a lower average Dmean of 15.9% and 14.9% respectively compared to VMAT

• Parameters derived from accumulated dose predicted a higher Grade ≥2 GI toxicity risk compared to planned dose and Quantec

• Parameters derived from accumulated dose predicted a lower Grade ≥2 GU toxicity risk compared to planned dose

## Introduction

Prostate cancer is a multifocal disease, whereby dominant intraprostatic lesions (DILs) are often the source of local failure after external beam radiotherapy, especially in patients with locally advanced high-risk prostate cancer (HR-PCa) (1). The incorporation of modern imaging scans such as multiparametric magnetic resonance imaging (mpMRI) in radiotherapy (RT) treatment planning enables the precise definition of DILs (2). mpMRI-defined DILs are highly correlated to the location and size of the histopathologically proven tumor on whole-mount prostatectomy specimens with high levels of clonogenic cell density (3). The delivery of an escalated dose to the whole prostate has reportedly improved biochemical disease-free survival (bDFS); however, the associated rates of gastrointestinal (GI) and genitourinary (GU) toxicity are higher (4, 5). The application of a simultaneous integrated boost (SIB) technique allows the delivery of an escalated dose to the DILs while respecting the planning constraints of the organs at risk (OARs) (6, 7).

In recent years, utilization of proton therapy in the clinical management of prostate cancer has rapidly increased (8). This is due to the distinct physical advantages of protons having a finite dose fall-off beyond the dose maximum (Bragg peak), which reduces the dose to OARs beyond the target (9). Although the majority of the published clinical data have reported the toxicity and efficacy of proton treatment on the prostate (10, 11), there is limited published work reporting on the utility of proton therapy in HR-PCa with pelvic lymph node (PLN) irradiation (8). Using proton therapy may be beneficial for these larger target volumes due to the capacity for greater sparing of proximal OARs as a result of increased conformality, translating to lower GI and GU toxicity compared to photon treatment (12). Intensity-modulated proton therapy (IMPT) using active pencil beam scanning technology can generate highly conformal SIB plans to facilitate dose escalation to the DILs in a single-phased regimen (13).

To the best of our knowledge, there is a scarcity of work focusing on the dosimetric and potential clinical outcomes between IMPT and volumetric modulated arc therapy (VMAT) with dose-escalated SIB to the DILs for HR-PCa with PLN using a hypofractionated schedule (14, 15). In this work, tumor control probability (TCP) and normal tissue complication probability (NTCP) metrics, in addition to the standard dose-volume (DV) metrics for plan assessment, were computed to provide a robust platform to determine the safety and effectiveness of DIL dose escalation. Additional NTCP metrics obtained from previously developed parameters derived from accumulated (*D*_A_-NTCP) and planned doses (*D*_p_-NTCP) were used.

The purpose of the study was to test the hypothesis that delivering an escalated dose to the DILs in HR-PCa patients using proton therapy can yield a higher TCP without a significant increase in NTCP to the OARs compared to the use of photon therapy.

## Materials and methods

In total, 10 HR-PCa patients treated between 2020 and 2021 using VMAT and a hypofractionated dose regimen with pretreatment mpMRI scans were retrospectively recruited for this study. Ethics approval was obtained by the Centralized Institutional Review Board (CIRB: 2020–2161).

### CT-simulation and mpMRI acquisition

Patients were simulated in a supine position with their hands on their chest using a leg immobilizer for reproducibility. Bladder filling instructions were provided by the radiation therapists (400–600 mL of water) to ensure a reasonably full bladder. In addition, patients were required to empty their bowels before CT stimulation and daily treatment. CT scans were acquired with a 2.5-mm-slice thickness (120 kVp, GE LightSpeed RT 16). Patients underwent mpMRI scans either on the same day as the CT simulation or the day after. mpMRI datasets consisting of T1-weighted (T1W), T2W, and diffusion-weighted imaging (DWI) scan sequences were acquired using the MAGNETOM Avanto (Siemens AG, Munich, Germany) 1.5 T scanner.

### DIL delineation

**Figure 1:**
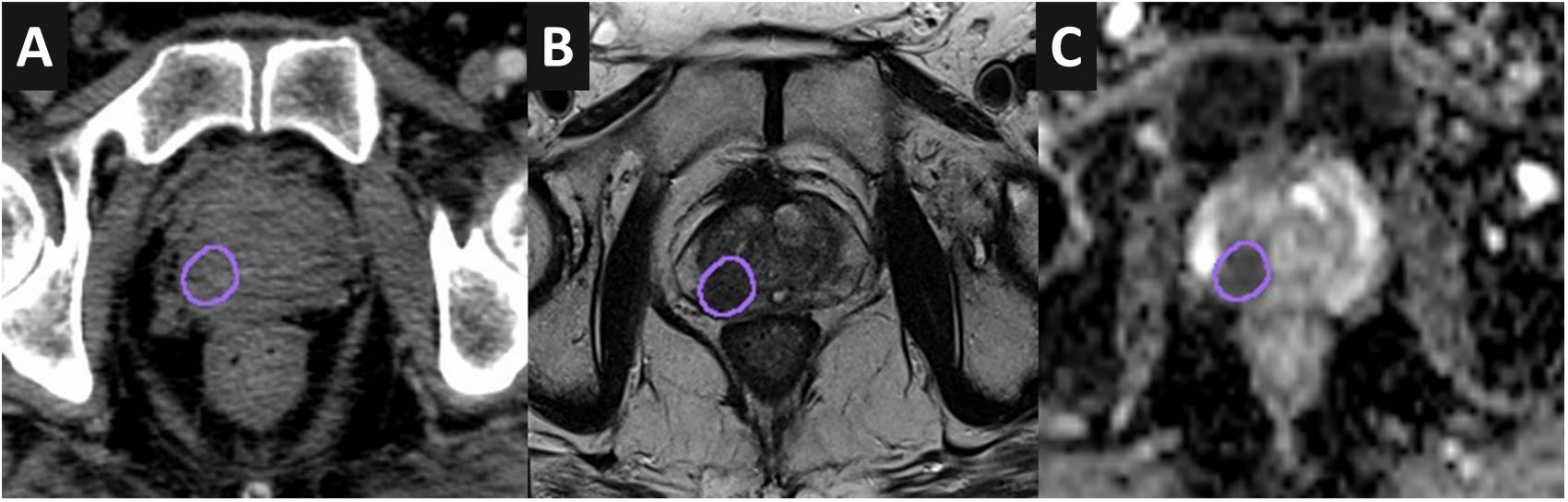
DIL contoured onto the CT scan **(A)**, T2W MRI **(B)**, and apparent diffusion coefficient map **(C)** of a case study.

DILs were identified and reported as per Prostate Imaging Reporting and Data System (PI-RADS) version 2.0 criteria (16) by the radiologist. Additional annotations were made with regard to the location and extension of the DILs on the radiological scans to facilitate radiation oncologists (RO) contouring. An experienced RO delineated the DILs on the planned CT scan after rigid registration (focusing on the prostate gland) was performed between the scans. DILs defined as the gross tumor volume were delineated with reference to the low intensity on T2W and hypointense on apparent diffusion coefficient (ADC) maps (17) (**Figure 1**). PTV_dil_ was generated from the DIL contour with a 2-mm expansion margin. No expansion margin was applied for DILs that were close to the urethra or rectum. The prostate and urethra were also contoured using the T2W MRI.

### Target definitions and dose prescription

**Figure 2:**
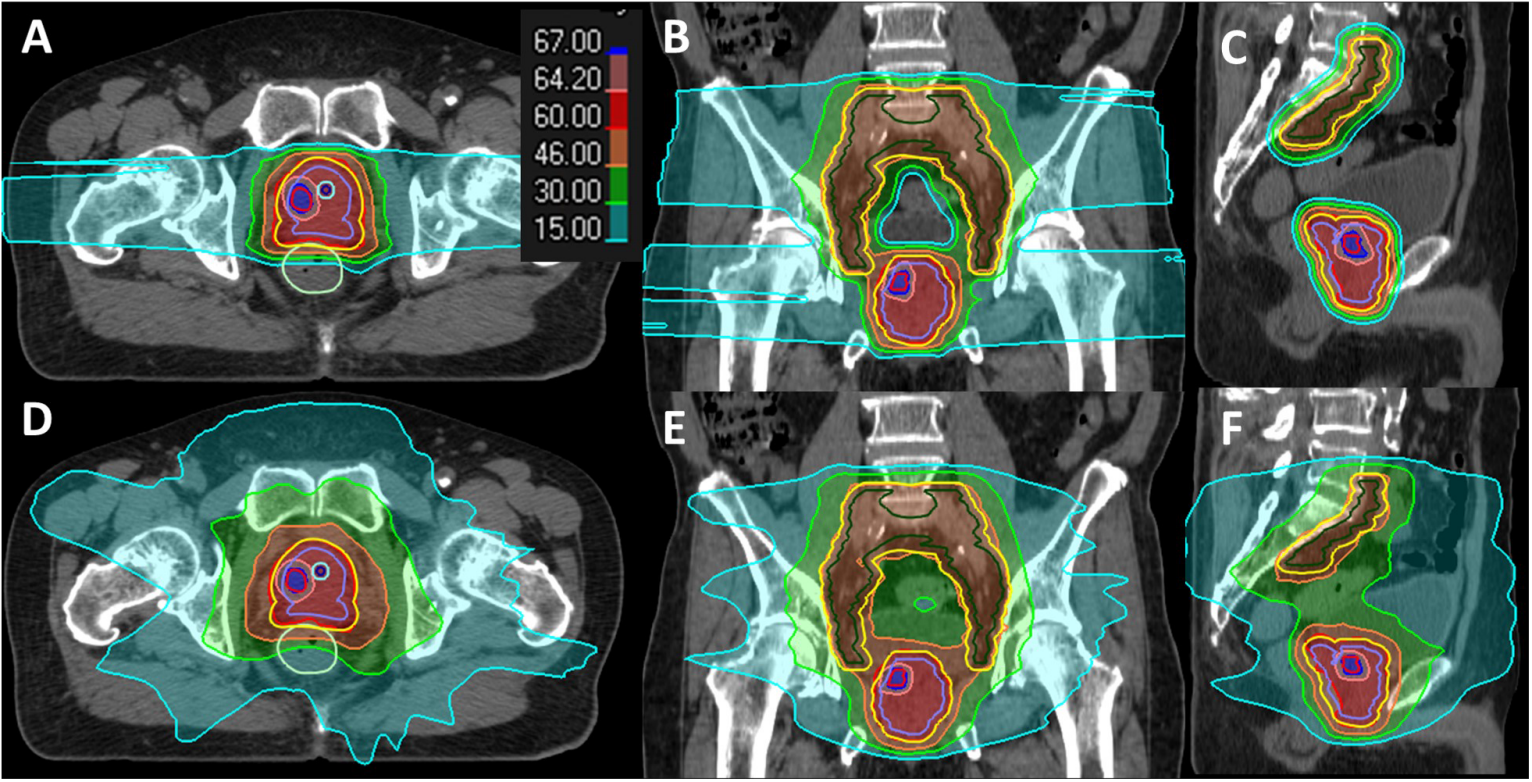
Dose distribution display in the color wash of a case study for IMPT **(A–C)** and VMAT **(D–F)** plans. The contours are color-coded as follows: DIL (red), CTVpsv (purple), CTVpln (green), PTVpsv + PTVpln (yellow), rectum (green), urethra (blue), and urethra PRV 2 mm (cyan).

The targets were defined as follows: clinical target volume with proximal seminal vesicles (CTVpsv): prostate plus proximal 2 cm or entire sv, depending on the level of sv involvement based on the MRI staging report (18). Planning target volume (PTV) psv: uniform 5 mm expansion margin from CTVpsv. CTVpln: L5-S1 junction or mid L5, encompassing bilateral common iliac, external iliac, internal iliac, presacral, and obturator nodes. PTVpln: uniform expansion of 7 mm from CTVpln. All CTVs and OARs were defined in accordance with the Radiation Therapy Oncology Group (RTOG) consensus guideline (**Figure 2**) (19). Target optimization for VMAT was based on PTV, while for IMPT, CTV was used. The prescribed dose was planned to deliver 60 Gy in 20 fractions to the CTVpsv/PTVpsv and 46 Gy to the CTVpln/PTVpln using a SIB technique. DV constraints were applied as per departmental guidelines (**Supplementary Table S1**), as well as an additional mandatory constraint for the urethra. As a constant relative biological effectiveness (RBE) of 1.1 was applied for the proton dose prescription, the term GyRBE was used in proton dose reporting.

### Treatment planning

To maintain plan consistency, two plans with DIL boost using IMPT and VMAT were generated for each patient by the same dosimetrist. A total of twenty plans (10 IMPT and 10 VMAT) were available for analysis. Both IMPT and VMAT were created using Raystation planning system 10A (RaySearch Laboratories AB, Stockholm, Sweden), and the Monte Carlo algorithm was used for dose calculation. All treatment plans were optimized based on the stated clinical goals in **Supplementary Table S1** (12, 20). For VMAT planning, two full arcs (clockwise to anti-clockwise rotations) using 10 MV energy with the collimator tilt at 10°–20° were utilized. The target coverage for VMAT was to have 95% of the PTV covering 95% of the prescription dose (PD).

IMPT using a multifield optimization (MFO) technique was utilized to facilitate dose escalation to the DILs. A proton beam arrangement consisting of two lateral beams and one posterior beam was employed. A central blocking structure was created to prevent spot placement in the contralateral PLN. A posterior block was included to minimize spot placement at the rectal region. IMPT was robustly optimized using a 5-mm setup and a 3.5% range of uncertainties to achieve the target coverage of 98% of the CTV, encompassing 98% of the PD. For proton planning in general, the target coverage requirement was more stringent than VMAT as the target assessment is based on CTV. The 5-mm setup uncertainty used in proton planning coincides with the CTV-to-PTV setup and organ motion expansion margins utilized in the photon environment. The additional 3.5% range uncertainty is necessary for proton planning as the proton beam is sensitive to the heterogeneity of the tissue transverse path (21). From a biological perspective, a robustly optimized treatment plan ensures that the CTV receives a consistent dose and mitigates the impact of uncertainties on the OARs in the simulated scenarios, thereby balancing the achieved TCP and NTCP. DV metrics were extracted based on the planning constraints for the target and OARs.

### Radiobiological models

A linear-quadratic (LQ)-Poisson model was used in this study (**Supplementary Material**, Eqs. A.1–4). The TCP parameters were adopted from a study conducted by Sachpazidis et al. (22) on 129 intermediate-HR-PCa patients with a median follow-up of 81.4 months and a biochemical relapse rate of 18%. TCP values were generated separately for the prostate minus DIL (prostate-DIL) and DIL due to the use of different model parameters, assuming the difference in tumor cells’ aggressiveness (6, 23). The low and very low α/β of 3 and 1.5 Gy were used to evaluate the different tumor sensitivity, as the exact value has yet to be determined (**Supplementary Table S2**) (24). A Lyman–Kutcher–Burman normal tissue complication probability (LKB-NTCP) model with an equivalent uniform dose (EUD) was employed for OAR estimation. Details on the generation of *D*_A_ and *D*_P_ and model formulation have previously been described (25, 26). LKB-NTCP parameters for the rectum and bladder were adopted from our previous work using *D*_A_ and *D*_P_ to fit the prospectively collated toxicity data for grade ≥ 2 based on 3-year post-RT follow-up time points. The maximum likelihood estimation method was used to estimate the best-fit parameters for LKB-NTCP (27). Additional rectum and bladder parameters obtained from published work (27–30) were also included for comparison (**Supplementary Table S3**). Similar to TCP calculations, extracted DV metrics were converted to equivalent doses at 2 Gy per fraction using the LQ model prior to NTCP analysis.

### Statistical analysis

Data normality was tested using the Shapiro–Wilk test. A paired *t*-test was used for DV analysis between IMPT and VMAT and reported as mean and standard deviations (SDs) as the data were normally distributed. A Wilcoxon signed-rank test was used for analyzing TCP and NTCP variables and documented as median and interquartile ranges (IQR) as the data were not normally distributed. Descriptive statistics were presented as mean and SDs as appropriate. Statistical analysis was conducted using Matlab (MathWorks, Inc., Natick, MA, USA, version 8.0) and IBM SPSS statistics (version 26.0, SPSS, Inc., Chicago, IL, USA). Two-sided *p*-values of < 0.05 were considered statistically significant.

## Results

In total, 10 patients with mpMRI-identified DILs were enrolled in this study. Five patients had two DILs detected, resulting in 15 DILs in this cohort. The mean prostate volume was 36.4 cm^3^, and the DILs had a mean volume of 1.1 cm^3^. Patient characteristics are summarized in **Table 1**.

**Table 1:** Patient-specific characteristics.

Patient characteristics	Frequency (*N* = 10)
Age (years; mean [± SD])	76 [± 6]
PSA at baseline (ng/mL; mean [± SD])	34.5 [± 25.3]
cT-stage (AJCC 8th edition)	
cT3a (%); cT3b (%)	2 (20%); 8 (80%)
Gleason score	
≤ 7 (%); > 7 (%)	2 (20); 8 (80)
No. of DIL	
1 (%); 2 (%)	5 (50); 5 (50%)
DIL volume (cm^3^; mean [± SD])	1.1 [± 0.7]
Prostate volume (cm^3^; mean [± SD])	36.4 [17.1]
DIL location	
Peripheral zone (%)	11 (73)
Transition Zone (%)	4 (27)

### Target DV and TCP

**Figure 3:**
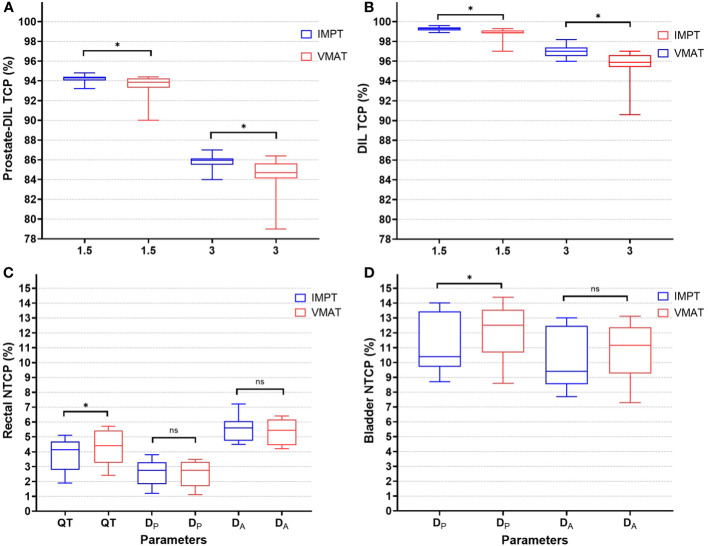
TCP (%) for prostate-DIL **(A)** and DIL **(B)** using α/β of 1.5 and 3 Gy for IMPT and VMAT. NTCP (%) for rectal **(C)** using parameters from Quantec (QT), planned (*D_P_*), and accumulated dose (*D_A_*) and bladder **(D)** for IMPT and VMAT. An asterisk (*) denotes a statistically significant difference between IMPT and VMAT on a paired *t*-test (*p* < 0.05). ns, not significant between the plans.

For DIL coverage, IMPT achieved on average a *D*mean of 68.1 Gy, while VMAT obtained a slightly lower *D*mean of 66.6 Gy (*p* < 0.05). The difference in mean dose between IMPT and VMAT was 1.4 Gy (*p* < 0.05). The maximum level of dose escalation achievable for the two modalities was equivalent, as the constraints for the dose-limiting structures for the urethra PRV 2 mm at *D*0.5 cm^3^ and rectum V60 Gy were not statistically significant (**Table 2**). For TCP modeling, IMPT generated a higher average median TCP with a narrower percentage range for prostate-DIL and DIL compared to VMAT (**Table 3**; **Figures 3A, B**).

**Table 2:** DV metrics and dose difference for the target and OARs generated from IMPT and VMAT.

Target/OAR	DV metrics	IMPT (GyRBE/%)	VMAT (GyRBE/%)	Dose diff (GyRBE/Gy/%)	*p*-value
DIL	*D*98%	65.5 (1.2)	64.5 (0.9)	1.0 (0.9)	< 0.05
	*D*95%	66.0 (1.0)	64.9 (0.8)	1.1 (0.9)	< 0.05
	*D*mean	68.1 (0.8)	66.6 (0.9)	1.4 (1.0)	< 0.05
	*D*0.03 cm^3^	69.8 (1.4)	68.0 (1.3)	1.8 (1.3)	< 0.05
CTVpsv	*D*98%	60.4 (0.3)	60.2 (0.2)	0.2 (0.4)	0.053
	*D*mean	62.8 (0.3)	62.5 (0.4)	0.3 (0.2)	< 0.05
	*D*1 cm^3^	68.2 (0.9)	66.9 (0.2)	1.3 (0.8)	< 0.05
CTVpln	*D*98%	46.5 (0.1)	46.1 (0.2)	0.4 (0.3)	< 0.05
PTVpsv	*D*95%	–	60.1 (0.1)	–	–
PTVpln	*D*95%	–	46.2 (0.2)	–	–
Rectum	V60 Gy	3.0 (1.1)	2.3 (0.8)	0.7 (1.1)	0.08
	V56 Gy	8.0 (3.0)	9.2 (3.0)	−1.2 (1.0)	< 0.05
	V52 Gy	10.3 (3.8)	13.7 (4.0)	−3.3 (2.0)	< 0.05
	V48 Gy	12.5 (4.1)	17.5 (4.5)	−5.0 (2.6)	< 0.05
	V40 Gy	16.6 (5.2)	26.6 (5.5)	−10 (3.1)	< 0.05
	V32 Gy	20.4 (6.0)	40.4 (7.8)	−20 (6.4)	< 0.05
	V24 Gy	24.8 (6.8)	61.2 (8.1)	−36.4 (9.3)	< 0.05
	*D*mean	15.0 (3.4)	31.0 (1.9)	−15.9 (2.4)	< 0.05
	*D*0.03 cm^3^	61.7 (0.4)	61.8 (0.4)	−0.04 (0.4)	0.80
Bladder	V60 Gy	5.4 (2.8)	4.9 (2.4)	0.5 (0.9)	0.11
	V48 Gy	13.4 (5.4)	17.8 (6.3)	−4.4 (3.5)	< 0.05
	V40 Gy	28.8 (5.3)	41.2 (8.3)	−12.4 (6.9)	< 0.05
	*D*mean	22.3 (3.0)	37.2 (3.2)	−14.9 (3)	< 0.05
	*D*0.03 cm^3^	64.8 (1.3)	64.4 (0.9)	0.4 (1.0)	0.20
Urethra	*D*0.03 cm^3^	63.4 (0.3)	63.3 (0.4)	0.1 (0.4)	0.20
Urethra PRV2 mm	*D*0.5 cm^3^	62.9 (0.2)	62.8 (0.5)	0.1 (0.3)	0.20
Femoral head	*D*mean	15.3 (0.8)	15.0 (1.6)	0.4 (1.9)	0.39

**Table 3:** TCP and NTCP for the targets and OARs.

	IMPT median (IQR; %)	VMAT median (IQR; %)	% Diff median	*p*-value
TCP for α/β: 1.5 Gy				
Prostate-DIL	94.3 (94.0–94.4)	93.9 (93.3–94.2)	0.9	< 0.05
DIL	99.3 (99.1–99.4)	98.9 (98.8–99.1)	0.2	< 0.05
TCP for α/β: 3 Gy				
Prostate-DIL	86.0 (85.5–86.1)	84.7 (84.1–85.6)	1.1	< 0.05
DIL	97.0 (96.5–97.4)	95.9 (95.4–96.6)	1.5	< 0.05
NTCP				
Rectal				
QT	4.2 (2.8–4.7)	4.4 (3.3–5.4)	−0.5	< 0.05
*D*_P_	2.7 (1.8–3.3)	2.7 (1.7–3.3)	0.1	0.59
*D*_A_	5.6 (4.1–4.5)	5.5 (4.4–6.2)	0.3	0.09
Bladder				
Burman	–	–	–	ns
*D*_P_	10.4 (9.7–13.4)	12.5 (10.7–13.5)	−0.9	< 0.05
*D*_A_	9.4 (8.5–12.4)	11.2 (9.2–12.3)	−0.6	0.11
Urethra	4.3 (4.1–4.5)	4.3 (3.9–4.4)	0.1	0.33
Femoral head	–	–	–	ns

TCP, tumor control probability; Pros-DIL, prostate cropped from DIL; % Diff, IMPT minus VMAT; LKB-NTCP, Lyman–Kutcher–Burman normal tissue complication probability; QT, Quantec; D_P_, dose planned; D_A_, dose accumulated; ns, not significant; IQR, interquartile range.

### Rectal and bladder DV and NTCP

For the rectum, dose ranges from V24 to V56 Gy was lower in IMPT compared to VMAT (*p* < 0.05). At the very high dose region, the dose to the rectum was not statistically significant between the two modalities (V60 Gy, *p* = 0.08). A substantial dose reduction at the low–intermediate-dose range in IMPT, with differences in mean dose at V24 Gy (IMPT minus VMAT: 36.4%, 9.3 Gy) and at V32 Gy (IMPT minus VMAT: 20%, 6.4 Gy), was observed (**Table 2**). In NTCP modeling, the rectal *n* value reported in Quantec (QT) and the derived *n* values from *D*_P_ and *D*_A_ ranging from 0.2 to 0.9 suggested an increased risk of high dose to a small rectal volume on GI toxicity (**Table 3**; **Figure 3C**). Rectal *D*_A_-NTCP displayed the highest grade ≥ 2 toxicity risk (IMPT: 5.6% vs. VMAT: 5.5%), followed by QT-NTCP (IMPT: 4.2% vs. VMAT: 4.4%), with *D*_P_-NTCP having the lowest risk estimation (IMPT and VMAT: 2.7%). For the bladder, apart from V60 Gy, a dose reduction was observed in the V40–48 Gy region in IMPT compared to VMAT (*p* < 0.05). The average bladder *D*mean for IMPT was 14.9% (3 Gy) lower compared to the VMAT (*p* < 0.05) (**Table 2**). Bladder NTCP was not significant from Burman-generated parameters for both modalities. *D*_P_-NTCP for IMPT was lower compared to VMAT (IMPT: 10.4% vs. VMAT: 12.5%, *p* < 0.05) (**Table 3**; **Figure 3D**).

### Urethra and femoral heads (DV and NTCP)

Dose to the hottest 0.03 cm^3^ of the urethra and D0.5 cm^3^ of the urethra PRV 2 mm for IMPT and VMAT were comparable (*p* = 0.33) as urethra was considered hard constraints during plan optimization. Similarly, NTCP for IMPT and VMAT were low and not statistically significant. An average mean dose to the femoral head for IMPT and VMAT had no statistically significant differences (*p* = 0.39) (**Table 2**). No result was generated for the NTCP for the femoral heads as the overall irradiated dose to the femoral heads was low.

## Discussion

Dose escalation to the mpMRI-defined DILs planned using protons and photons on patients with HR-PCa with PLN was conducted. Apart from the DV-based metrics, TCP and NTCP values were also generated to estimate the probability of achieving the optimal therapeutic ratio by maximizing the TCP without compromising on the NTCP of the OARs. To ensure the plans were clinically treatable (without having excessive splashes of high doses within the prostate), a *D*mean criterion of 67 Gy (BED: 216.6, α/β: 1.5 Gy) was included as the planning goal. This was estimated to have a BED of greater than 200 Gy for the DILs, as recommended by published clinical trials on performing DIL focal boost (20, 31).

### Target DV and TCP

IMPT met the required CTV coverage of *D*95% > 95% at worst-case scenarios (CTVpsv; median: 59.46 GyRBE, CTVpln: 45.3 GyRBE). Thus, the plans were robust enough to be treated clinically (**Supplementary Table S4**). This study was designed to perform a safe dose escalation using IMPT and VMAT by setting a mandatory constraint on the surrounding OARs. Notably, urethra PRV 2 mm (*D*0.5 cm^3^ < 64 Gy) and rectum (V60 Gy < 5%) were the main dose-limiting constraints. A similar strategy was adopted in the PIVOTALboost (ISRCTN80146950) trial, whereby the dose of the DIL was limited to 70 Gy over 20 fractions (20). Both IMPT and VMAT were able to achieve high TCP results for prostate-DIL and DILs, with IMPT obtaining a moderately higher TCP compared to VMAT (*p* < 0.05) (**Table 3**). As the DILs are regarded as high-risk targets, correlating to the likelihood of disease relapse, escalating the dose to an estimated 11.7% above the prescription dose could potentially attain a higher disease control rate of 97% (α/β: 3 Gy) and 99.3% (α/β: 1.5 Gy) for IMPT.

The higher average median TCP achieved for IMPT (*p* < 0.05) remained consistently high for all the plans compared to VMAT for the α/β ratio of 1.5 Gy and 3 Gy (**Figure 3**). This could be attributed to the optimal dosimetric properties of the proton beam, which have the ability to create a sharp dose gradient at the distal end of the beam (32). Similar approaches have been demonstrated by Uzan et al. (33) on 11 HR-PCa patients using conventional fractions (74 Gy in 37 fractions), treating only the prostate gland with intensity-modulated radiotherapy. By limiting the DILs to 86 Gy (BED: 219 Gy), while maintaining the rectal NTCP to 5%–6%, an increase in TCP from 71% to 83.6% was observed, with no grade 3 events reported at 36 months post-RT. Conversely, some studies adopted a “maximum achievable dose” escalation strategy by using a step-by-step dose-incremental approach to enhance the dose to the DILs while keeping the NTCP at an acceptable level (32). However, the use of this approach must be carefully balanced between potential increase in TCP and toxicity risk, as the urethra was not routinely contoured and considered a dose-limiting organ in some studies (13, 20).

### Rectal DV and NTCP

For the rectal DV metrics, V60 Gy for both modalities was similar due to the close proximity of this organ to the target. However, as the proton beam deposited virtually no dose beyond the target, significant sparing of the rectum was observed in the low-intermediate dose region (8). The increase in linear energy transfer (LET) at the distal edge of the proton beam has the potential to cause an increase in toxicity (34). The use of two laterally opposed beams and the modulation of beam energy and intensity during plan optimization were strategies used to mitigate the risk of increased toxicity at the distal edge (35).

It is still unclear if the extensive reduction in the low-intermediate dose will correspond to a lower risk of GI toxicity. However, a multi-institutional prospective trial database review of a subset of PCa patients with PLN irradiation treated using proton therapy reported low rates of acute (grade 2: 2%, grade 3: 0) and late (grade 2: 2%, grade 3: 1%) GI toxicity (36). The reduction in dose received by the rectum at the high dose range in IMPT compared to VMAT (V56 Gy and V52 Gy, *p* < 0.05) could potentially be translated into a lower risk of patients having grade ≥2 GI toxicity (27).

For rectal NTCP modeling, Cicchetti et al. (37) reported on late rectal bleeding for 1,633 prostate cancer patients with a minimum of 3-year follow-up using various EUD-based NTCP parameters and concluded that higher *n* values of 0.18 and 0.24 better predict late grades ≥2 and 3 GI toxicity. The study demonstrated that the rectum might not behave as predominantly serial-like but has some parallel-like properties, whereby the intermediate–high-dose region might increase the risk of GI toxicity. The use of IMPT in this study demonstrates a reduction in rectal dose for the intermediate–high-dose range (V24–56 Gy, *p* < 0.05) and thus has the potential to minimize the risk of GI toxicity. The risk of having grade ≥2 GI toxicity is generally low, whereby both IMPT and VMAT-derived *D*_A_-NTCP values achieved comparable toxicity risk estimations as a mandatory rectal V60 Gy < 5% constraint was applied. In considering rectal *D*_A_-NTCP, there was a 2.9% and 2.8% increase in the risk of predicting grade ≥2 GI toxicity compared to *D*_P_-NTCP for IMPT and VMAT, respectively. This provides a good reference guide for clinicians when devising dose-escalation protocols and selecting treatment modalities, as interfraction rectal motion should be considered because the impact on the risk of toxicity could be more pronounced (27).

### Bladder DV and NTCP

For the bladder DV analysis, a significant reduction in the average mean dose was observed in IMPT, which could potentially be translated into a reduction in acute and late GU toxicity (36). Choo et al. (12) reported acceptable late GU toxicity in their prospective trial, with none of the 56 patients experiencing grade 3 GU toxicity with PLN treated with proton therapy. For NTCP modeling, given the similar *D*_50_ and *m* values from *D*_P_- and *D*_A_-generated biological parameters, statistical significance was observed between IMPT and VMAT. This could imply that the *n* value for the bladder could be closer to 0.14, indicating the serial-parallel-like behavior of the bladder to radiation (27).

### Urethra DV and NTCP

Due to the high incidences of reported late GU toxicity as well as urethra strictures, recent randomized trials have started to contour and include urethra constraints to minimize toxicity (32). The availability of the mpMRI scans at a similar time point as the planned CT scans in this study allows for accurate contouring of the urethra. An additional urethra 2 mm PRV margin with a mandatory *D*0.5 cm^3^ < 64 Gy dose limit further reduced the high-dose spillage to the urethra, especially for DILs that were located close to the urethra (20). In line with the objective of safe-dose escalation, after applying the mandatory urethra dose limit, both IMPT and VMAT achieved a low NTCP of 4.3%. A moderately higher DIL mean dose was associated with the risk of urethra stricture with IMPT compared to VMAT (IMPT: 68.1 GyRBE vs. VMAT: 66.6 Gy, *p* < 0.05).

### Femoral head DV and NTCP

Despite the use of two lateral-opposed beams in IMPT, the average *D*mean for the femoral heads was low and comparable to VMAT (*p* = 0.39). Conversely, a study reported by Whitaker et al. (38), comparing the dosimetric difference between IMPT and VMAT on PCa patients with PLN treatment, obtained a higher femoral head *D*mean for IMPT (IMPT: 18.2 GyRBE vs. VMAT: 14.8 Gy, *p* < 0.05). The lower femoral head *D*mean achieved in this study could be due to the use of an additional posterior proton beam. The concern of introducing a posterior beam due to the presence of rectal uncertainties was addressed by adding a blocking structure to prevent spot placement around the rectal region. This technique has effectively reduced the femoral head doses even with DIL boosting (32). NTCP for a femoral head was not significant as the dose received was very low.

Firstly, this study only included 10 patients. This study explored our initial experience by comparing photon and proton planning techniques, including the application of biological indices to predict potential treatment outcomes and toxicity risks for patients with HR-PCa with PLN irradiation. The generalizability of these results needs to be confirmed in a larger cohort study. Other clinical aspects such as disease-free survival, overall survival, and toxicity rates based on patient-reported outcomes can be incorporated into the design of the risk-benefit profile in a prospective manner as an extension of this study. Secondly, although biological modeling provides practical metrics for plan comparison and optimization, the results might not be a true representation of actual clinical outcomes (39). The accuracy of TCP/NTCP models is highly dependent on the selection of models, the derived parameters, and the associated clinical endpoints (40). To note, the majority of the parameters (Quantec and Burman) used in NTCP modeling were mainly based on three-dimensional conformal techniques. To minimize these uncertainties, well-established TCP and NTCP models and parameters were selected in this study. Moreover, the addition of previously developed NTCP parameters for the rectum and bladder based on similar patient cohorts and dose distribution improves the reliability of GI and GU toxicity estimates (27). Furthermore, taking into consideration the impact of bladder and rectal motion in these parameters increases the accuracy of toxicity predictions, which has not been reported in the literature. Lastly, the reliability of using TCP/NTCP models on proton therapy has been under ongoing investigation due to the increased application of proton therapy to more disease subsites and the availability of more mature published clinical data (41). Currently, the clinical application of proton therapy assumes a constant RBE of 1.1, which will have minimal impact on the α/β, and hence the dose–response curve remains comparable for both photons and protons (42). Further research is still required in this aspect to fully tap into the benefits of incorporating biological modeling in clinical decision-making.

As half of the study cohort had two DILs, this analysis was able to address the challenging aspects of attempting to achieve an escalated dose of the DILs while meeting the mandatory constraints for the OARs. As IMPT is able to offer a highly conformal dose distribution with excellent dose fall-off properties, all the planned cases have achieved a higher minimum TCP with comparable NTCP to VMAT. Lastly, the incorporation of motion-inclusive NTCP parameters derived from similar patient cohorts further improves the toxicity predictions. Moving forward, apart from increasing the sample size to enhance the reliability of the results, more work is still needed to investigate the utilization of biological models in determining clinical outcomes, especially in the field of proton therapy.

In conclusion, this preliminary study has demonstrated that dose escalation to the DILs using IMPT is able to achieve a higher TCP while keeping the NTCP comparable to the VMAT. Rectal *D*_A_-NTCP displayed a greater predicted GI toxicity compared to *D*_P_- and QT-derived parameters. In contrast, bladder *D*_A_-NTCP has a slightly lower predicted toxicity compared to *D*_P_-NTCP. The inclusion of interfraction organ motion improves the reliability of the predicted toxicity.

## Data availability statement

The original contributions presented in the study are included in the article/**Supplementary Material**. Further inquiries can be directed to Ashley.ong@monash.edu.

## Author contributions

AO wrote the initial draft of the manuscript. All authors participated in the editing and review of the manuscript. All authors contributed to the article and approved the submitted version.

## References

[1] Chopra S Toi A Taback N Evans A Haider MA Milosevic M. Pathological predictors for site of local recurrence after radiotherapy for prostate cancer. Int J Radiat Oncol Biol Phys (2012) 82:e441–8. doi: 10.1016/j.ijrobp.2011.05.035 22284038

[2] Girometti R Cereser L Bonato F Zuiani C. Evolution of prostate MRI: from multiparametric standard to less-is-better and different-is better strategies. Eur Radiol Exp (2019) 3:5–19. doi: 10.1186/s41747-019-0088-3 30693407 PMC6890868

[3] Borren A Groenendaal G Moman MR Boeken Kruger AE van Diest PJ van Vulpen M. Accurate prostate tumour detection with multiparametric magnetic resonance imaging: Dependence on histological properties. Acta Oncol (2014) 53:88–95. doi: 10.3109/0284186X.2013.837581 24041257

[4] Macias VA Barrera-Mellado I. Ultra-hypofractionated radiation therapy for unfavourable intermediate-risk and high-risk prostate cancer is safe and effective: 5-year outcomes of a phase II trial. BJU Int (2020) 125:215–25. doi: 10.1111/bju.14925 PMC7003804 31614071

[5] Rodda S Tyldesley S Morris WJ Keyes M Halperin R Pai H. ASCENDE-RT: an analysis of treatment-related morbidity for a randomized trial comparing a low-dose-rate brachytherapy boost with a dose-escalated external beam boost for high- and intermediate-risk prostate cancer. Int J Radiat Oncol Biol Phys (2017) 98:286–95. doi: 10.1016/j.ijrobp.2017.01.008 28433432

[6] Laughlin BS Silva AC Vora SA Keole SR Wong WW Schild MH. Long-term outcomes of prostate intensity-modulated radiation therapy incorporating a simultaneous intra-prostatic MRI-directed boost. Front Oncol (2022) 12:921465. doi: 10.3389/fonc.2022.921465 36033460 PMC9399820

[7] Kerkmeijer LGW Groen VH Pos FJ Haustermans K Monninkhof EM Smeenk RJ. Focal boost to the intraprostatic tumor in external beam radiotherapy for patients with localized prostate cancer: results from the FLAME randomized phase III trial. J Clin Oncol (2021) 39:787–96. doi: 10.1200/jco.20.02873 33471548

[8] Royce TJ Efstathiou JA. Proton therapy for prostate cancer: A review of the rationale, evidence, and current state. Urol Oncol (2019) 37:628–36. doi: 10.1016/j.urolonc.2018.11.012 30527342

[9] Mohan R. A review of proton therapy – Current status and future directions. Precis Radiat Oncol (2022) 6:164–76. doi: 10.1002/pro6.1149 PMC9499036 36160180

[10] Arimura T Yoshiura T Matsukawa K Kondo N Kitano I Ogino T. Proton beam therapy alone for intermediate- or high-risk prostate cancer: an institutional prospective cohort study. Cancers (Basel) (2018) 10:116–31. doi: 10.3390/cancers10040116 PMC5923371 29642619

[11] Mendenhall NP Hoppe BS Nichols RC Mendenhall WM Morris CG Li Z. Five-year outcomes from 3 prospective trials of image-guided proton therapy for prostate cancer. Int J Radiat Oncol Biol Phys (2014) 88:596–602. doi: 10.1016/j.ijrobp.2013.11.007 24521677

[12] Choo R Hillman DW Mitchell C Daniels T Vargas C Rwigema JC. Late toxicity of moderately hypofractionated intensity modulated proton therapy treating the prostate and pelvic lymph nodes for high-risk prostate cancer. Int J Radiat Oncol Biol Phys (2023) 115:1085–94. doi: 10.1016/j.ijrobp.2022.11.027 36427645

[13] Moteabbed M Harisinghani M Paganetti H Trofimov A Lu H-M Efstathiou JA. Proton vs. photon radiotherapy for MR-guided dose escalation of intraprostatic lesions. Acta Oncol (2021) 60:1283–90. doi: 10.1080/0284186X.2021.1947523 34282708

[14] Syndikus I Cruickshank C Staffurth J Tree A Henry A Naismith O. PIVOTALboost: A phase III randomised controlled trial of prostate and pelvis versus prostate alone radiotherapy with or without prostate boost (CRUK/16/018). Clin Transl Radiat Oncol (2020) 25:22–8. doi: 10.1016/j.ctro.2020.08.003 PMC7508714 32995575

[15] Catton CN Lukka H Gu CS Martin JM Supiot S Chung PWM. Randomized trial of a hypofractionated radiation regimen for the treatment of localized prostate cancer. J Clin Oncol (2017) 35:1884–90. doi: 10.1200/jco.2016.71.7397 28296582

[16] Steiger P Thoeny HC. Prostate MRI based on PI-RADS version 2: how we review and report. Cancer Imaging (2016) 16:9–18. doi: 10.1186/s40644-016-0068-2 27067275 PMC4828836

[17] Van Houdt PJ Ghobadi G Schoots IG Heijmink S de Jong J van der Poel HG. Histopathological features of MRI-invisible regions of prostate cancer lesions. J Magn Reson Imaging (2020) 51:1235–46. doi: 10.1002/jmri.26933 31588646

[18] Park SY Cho NH Jung DC Oh YT. Prostate imaging-reporting and data system version 2: beyond prostate cancer detection. Korean J Radiol (2018) 19:193–200. doi: 10.3348/kjr.2018.19.2.193 29520176 PMC5840047

[19] Lawton CA Michalski J El-Naqa I Buyyounouski MK Lee WR Menard C. RTOG GU Radiation oncology specialists reach consensus on pelvic lymph node volumes for high-risk prostate cancer. Int J Radiat Oncol Biol Phys (2009) 74:383–7. doi: 10.1016/j.ijrobp.2008.08.002 PMC2905150 18947938

[20] Onjukka E Uzan J Baker C Howard L Nahum A Syndikus I. Twenty fraction prostate radiotherapy with intra-prostatic boost: results of a pilot study. Clin Oncol (2017) 29:6–14. doi: 10.1016/j.clon.2016.09.009 27692920

[21] Schreuder AN Shamblin J. Proton therapy delivery: what is needed in the next ten years? Br J Radiol (2020) 93:20190359. doi: 10.1259/bjr.20190359 31692372 PMC7066946

[22] Sachpazidis I Mavroidis P Zamboglou C Klein CM Grosu A-L Baltas D. Prostate cancer tumour control probability modelling for external beam radiotherapy based on multi-parametric MRI-GTV definition. Radiat Oncol (2020) 15:242–54. doi: 10.1186/s13014-020-01683-4 PMC7574270 33081804

[23] Zamboglou C Wieser G Hennies S Rempel I Kirste S Soschynski M. MRI versus (6)(8)Ga-PSMA PET/CT for gross tumour volume delineation in radiation treatment planning of primary prostate cancer. Eur J Nucl Med Mol Imaging (2016) 43:889–97. doi: 10.1007/s00259-015-3257-5 26592938

[24] Van Leeuwen CM Oei AL Crezee J Bel A Franken NAP Stalpers LJA. The alfa and beta of tumours: a review of parameters of the linear-quadratic model, derived from clinical radiotherapy studies. Radiat Oncol (2018) 13:96–107. doi: 10.1186/s13014-018-1040-z 29769103 PMC5956964

[25] Ong A Knight K Panettieri V Dimmock M Tuan JKL Tan HQ. Application of an automated dose accumulation workflow in high-risk prostate cancer - validation and dose-volume analysis between planned and delivered dose. Med Dosim (2022) 47:92–7. doi: 10.1016/j.meddos.2021.09.004 34740517

[26] Ong ALK Knight K Panettieri V Dimmock M Tuan JKL Tan HQ. Dose-volume analysis of planned versus accumulated dose as a predictor for late gastrointestinal toxicity in men receiving radiotherapy for high-risk prostate cancer. Phys Imaging Radiat Oncol (2022) 23:97–102. doi: 10.1016/j.phro.2022.07.001 35879938 PMC9307677

[27] Ong ALK Knight K Panettieri V Dimmock M Tuan JKL Tan HQ. Predictive modelling for late rectal and urinary toxicities after prostate radiotherapy using planned and delivered dose. Front Oncol (2022) 12:1084311. doi: 10.3389/fonc.2022.1084311 36591496 PMC9800591

[28] Michalski JM Gay H Jackson A Tucker SL Deasy JO. Radiation dose-volume effects in radiation-induced rectal injury. Int J Radiat Oncol Biol Phys (2010) 76:S123–S9. doi: 10.1016/j.ijrobp.2009.03.078 PMC3319467 20171506

[29] Emami B Lyman J Brown A Coia L Goitein M Munzenrider JE. Tolerance of normal tissue to therapeutic irradiation. Int J Radiat Oncol Biol Phys (1991) 21:109–22. doi: 10.1016/0360-3016(91)90171-y 2032882

[30] Panettieri V Rancati T Onjukka E Ebert MA Joseph DJ Denham JW. External validation of a predictive model of urethral strictures for prostate patients treated with HDR brachytherapy boost. Front Oncol (2020) 10:910. doi: 10.3389/fonc.2020.00910 32596153 PMC7300245

[31] Tree AC Satchwell L Alexander E Blasiak-Wal I deSouza NM Gao A. Standard and hypofractionated dose escalation to intraprostatic tumor nodules in localized prostate cancer: 5-year efficacy and toxicity in the DELINEATE trial. Int J Radiat Oncol Biol Phys (2022). 15:305–16. doi: 10.1016/j.ijrobp.2022.09.058 36150450

[32] Wang T Zhou J Tian S Wang Y Patel P Jani AB. A planning study of focal dose escalations to multiparametric MRI-defined dominant intraprostatic lesions in prostate proton radiation therapy. Br J Radiol (2020) 93:1–10. doi: 10.1259/bjr.20190845 PMC7066949 31904261

[33] Uzan J Nahum A Syndikus I. Prostate dose-painting radiotherapy and radiobiological guided optimisation enhances the therapeutic ratio. Clin Oncol (2016) 28:165–70. doi: 10.1016/j.clon.2015.09.006 26482453

[34] Underwood T Paganetti H. Variable proton relative biological effectiveness: how do we move forward? Int J Radiat Oncol Biol Phys (2016) 95:56–8. doi: 10.1016/j.ijrobp.2015.10.006 27084627

[35] Marteinsdottir M Paganetti H. Applying a variable relative biological effectiveness (RBE) might affect the analysis of clinical trials comparing photon and proton therapy for prostate cancer. Phys Med Biol (2019) 64:115027. doi: 10.1088/1361-6560/ab2144 31082810

[36] Chuong MD Hartsell W Larson G Tsai H Laramore GE Rossi CJ. Minimal toxicity after proton beam therapy for prostate and pelvic nodal irradiation: results from the proton collaborative group REG001-09 trial. Acta Oncol (2018) 57:368–74. doi: 10.1080/0284186x.2017.1388539 29034790

[37] Cicchetti A Fiorino C Ebert MA Iacovacci J Kennedy A Joseph DJ. Validation of prediction models for radiation-induced late rectal bleeding: evidence from a large pooled population of prostate cancer patients. Radiother Oncol (2023) 183:109628. doi: 10.1016/j.radonc.2023.109628 36934896

[38] Whitaker TJ Routman DM Schultz H Harmsen WS Corbin KS Wong WW. IMPT versus VMAT for pelvic nodal irradiation in prostate cancer: A dosimetric comparison. Int J Part Ther (2019) 5:11–23. doi: 10.14338/IJPT-18-00048.1 PMC6874187 31788504

[39] Bertolet A Carabe-Fernandez A. Clinical implications of variable relative biological effectiveness in proton therapy for prostate cancer. Acta Oncol (2020) 59:1171–7. doi: 10.1080/0284186X.2020.1762928 32427011

[40] Mesbahi A Rasouli N Mohammadzadeh M Nasiri Motlagh B Ozan Tekin H. Comparison of radiobiological models for radiation therapy plans of prostate cancer: three-dimensional conformal versus intensity modulated radiation therapy. J BioMed Phys Eng (2019) 9:267–78. doi: 10.31661/jbpe.v9i3Jun.655 PMC6613163 31341872

[41] Sørensen BS Pawelke J Bauer J Burnet NG Dasu A Høyer M. Does the uncertainty in relative biological effectiveness affect patient treatment in proton therapy? Radiother Oncol (2021) 163:177–84. doi: 10.1016/j.radonc.2021.08.016 34480959

[42] Paganetti H. Relative biological effectiveness (RBE) values for proton beam therapy. Variations as a function of biological endpoint, dose, and linear energy transfer. Phys Med Biol (2014) 59:R419–72. doi: 10.1088/0031-9155/59/22/r419 25361443

