# Supplementary material for: Proton versus photon therapy for high-risk prostate cancer with dose escalation of dominant intraprostatic lesions: a preliminary planning study
Source: Front Oncol. 2023 Nov 8;13:1241711. doi: 10.3389/fonc.2023.1241711 (PMC10663272; doi:10.3389/fonc.2023.1241711)
Supplement: Supplementary file 1 [file Table_1.docx]

Supplementary Material

Proton versus photon therapy for high-risk prostate cancer with dose escalation of dominant intraprostatic lesions: A preliminary planning study

**Ashley Li Kuan Ong^*^, Kellie Knight, Vanessa Panettieri, Mathew Dimmock, Jeffrey Kit Loong Tuan, Hong Qi Tan, Caroline Wright**

*** Correspondence:** Ashley Ong: [Ashley.ong.l.k@nccs.com.sg](mailto:Ashley.ong.l.k@nccs.com.sg)

TABLE S1. Target and OARs clinical goals for moderately hypofractionated regimen. Dose reported in GyRBE for IMPT and Gy for VMAT.

| **Target/OAR** | **DV-metrics** | **Goal (Mandatory)** | **Goal (Ideal)** |
| --- | --- | --- | --- |
| DIL | Dmean |  | 67 Gy |
| CTVpsv | D98% | 58.8 Gy |  |
| CTVpln | D98% | 45.08 Gy |  |
| PTVpsv (VMAT) | D95% | 57 Gy |  |
| PTVpln (VMAT) | D95% | 43.7 Gy |  |
| Rectum | V60Gy | 5% | 3% |
|  | V56Gy | 15 | - |
|  | V52Gy | 30 | - |
|  | V48Gy | 35 | 27 |
|  | V40Gy | 60 | 38 |
|  | V32Gy | 65 | 51 |
|  | V24Gy | 80 | 70 |
| Bladder | V60Gy | 35 | 5 |
|  | V48Gy | 25 | 50 |
|  | V40Gy | 50 | - |
| Urethra | D0.03cc | 64 | - |
| Urethra PRV 2mm | D0.5cc | 64 | - |
| Femoral head | V40Gy | 50 | 5 |
|  | D5cc | - | - |
|  | Dmean | - | - |

Abbreviations: OARs = organs at risk, IMPT = intensity modulated proton therapy, VMAT = volumetric modulated arc therapy, DV = dose-volume, PRV = planning risk volume.

Linear-quadratic (LQ) Poisson tumor control probability model (1).

**LQ Poisson formulation is as follows:**

$P (D)=e^{{-N}_{0}e^{-\alpha nd-\beta nd^{2}}} =e^{{-e}^{e\gamma-\left( \frac{nd}{D_{50}} \right)\left( e\gamma-\ln\ln2 \right)}}$ Eq. (A.1)

Where P(D) is the tumour response probability derived from a dose distribution, N_0_ is the initial number of clonogenic tumor cells, d is defined as the fractional dose, n is the number of treatment fractions, α and β are the linear quadratic parameters for cell killing, $\gamma$ is the slope of the dose-response curve and D_50_ is the dose required to achieve a fifty percent response probability. Extracted DV data were converted to equivalent dose of 2 Gy per fraction as expressed in Eq. (A.2) prior to the application of the equation to coincide with the fixed fractionated dose derived from the model (2).

${EQD}_{2}= \frac{nd\left( 1+\frac{d}{\frac{\alpha}{\beta}} \right)}{1+\frac{2}{\frac{\alpha}{\beta}}}$ Eq. (A.2)

${EQD}_{2,i}=\frac{\sum_{k=1}^{n} \left\{ d_{k,i}\left( 1+\frac{d_{k,i}}{\frac{\alpha}{\beta}} \right) \right\}}{1+\frac{2}{\frac{\alpha}{\beta}}}$ Eq. (A.3)

Eq. (A.3) takes into consideration the inhomogeneity of the dose distribution in the target volume whereby EQD_2, i_ is the EQD_2_ of a voxel element, i is the voxel number, k is the fraction number and d_k, i_ is the dose to voxel i obtained from fraction k. Combination of eq. (A.1) and (A.3):

$TCP(D)=\prod_{i=1}^{M} \left[ exp\left( -N_{0}exp\left( \sum_{k=1}^{n} \left\{ -\alpha d_{k,i}-\beta d_{k,i}^{2} \right\} \right) \right) \right]^{{v_{i}}/{v_{ref}}}$

$=\prod_{i=1}^{N_{v}} \left[ exp\left( -exp \left[ e\gamma- \frac{{EQD}_{2,i}}{D_{50}}\left( e\gamma-\ln\left( \ln\left( 2 \right) \right) \right) \right] \right) \right]^{{v_{i}}/{v_{ref}}}$ Eq. (A.4)

Where v_i_ is the voxel volume, v_ref_ is the total reference volume, M is the total number of voxels, and N_V_ is the total number of EQD_2_ derived from all voxels.

TABLE S2. TCP parameters used for LQ-Poisson model considering the α/β ratio of 1.5 and 3 Gy for the DIL and prostate-DIL.

| **Target** | **α/β, Gy** | $\boldsymbol{D}_{\boldsymbol{50}}$**, Gy** | $\boldsymbol{\gamma}$ |
| --- | --- | --- | --- |
| DIL_1.5_ | 1.5 | 68.1 | 4.5 |
| Prostate – DIL_1.5_ | 1.5 | 66.8 | 3.8 |
| DIL_3_ | 3 | 68.1 | 4.5 |
| Prostate – DIL_3_ | 3 | 66.8 | 3.8 |

Abbreviations: TCP = tumor control probability, LQ = linear quadratic, DIL = dominant intraprostatic lesion, D_50_= dose resulting in 50% probability of complication in a uniformly irradiated tissue, $\gamma$ = dimensionless parameter; maximum normalized value of the dose-response gradient.

TABLE S3. LKB-NTCP parameters with associated clinical endpoints.

| **Organ** | $\mathbf{n}$ | $\mathbf{m}$ | $\mathbf{D}_{\mathbf{50}}$ **(Gy)** | $\boldsymbol{\alpha}/\boldsymbol{\beta}$ **(Gy)** | **End point** |
| --- | --- | --- | --- | --- | --- |
| **Rectum** | | | | | |
| Quantec (QT) | 0.09 | 0.13 | 76.9 | 3 | Grade ≥ 2 late rectal bleeding (3) |
| D_P_ | 0.04 | 0.06 | 74.6 | 3 | Grade ≥ 2 GI toxicity at 3 years (4) |
| D_A_ | 0.02 | 0.07 | 77.9 | 3 | Grade ≥ 2 GI toxicity at 3 years (4) |
| **Bladder** | | | | | |
| Burman | 0.5 | 0.11 | 80 | 3 | Bladder contracture/ volume loss (5) |
| D_P_ | 0.14 | 0.27 | 81.9 | 3 | Grade ≥ 2 toxicity at 3 years (4) |
| D_A_ | 0.12 | 0.24 | 81.7 | 3 | Grade ≥ 2 toxicity GU at 3 years (4) |
| **Urethra** | 0.3 | 0.23 | 116.7 | 5 | Stricture requiring urethrotomy within 4 years after RT completion (6) |
| **Femoral head** | 0.25 | 0.12 | 65 | 3 | necrosis (5) |

Abbreviations: NTCP= normal tissue complication probability; m= dose-response parameter; n= volume effect parameter; D_50_= dose resulting in 50% probability of complication in a uniformly irradiated tissue.

TABLE S4. Robust analysis of the perturbed dose for IMPT based on voxel-wise minimum CTV coverage (D95% >95% of the prescription dose) with simulated worst-case scenarios for setup and range uncertainties.

|  | 5 mm and 3.5% | 5 mm and 3.5% |
| --- | --- | --- |
| Case | **CTVpsv, GyRBE** | **CTVpln, GyRBE** |
| 1 | 58.7 | 44.8 |
| 2 | 58.5 | 44.8 |
| 3 | 59.4 | 44.9 |
| 4 | 59.6 | 45.4 |
| 5 | 59.9 | 45.4 |
| 6 | 58.7 | 44.8 |
| 7 | 59.7 | 45.3 |
| 8 | 59.6 | 45.3 |
| 9 | 59.9 | 45.5 |
| 10 | 60.2 | 45.5 |
| Median ± IQR | 59.46 (58.7-59.9) | 45.3 (44.8-45.4) |

REFERENCES

[1] Sachpazidis I, Mavroidis P, Zamboglou C, Klein CM, Grosu A-L, Baltas D. Prostate cancer tumour control probability modelling for external beam radiotherapy based on multi-parametric MRI-GTV definition. *Radiat Oncol*. (2020) 15:242-54. doi: 10.1186/s13014-020-01683-4

[2] Allen Li X, Alber M, Deasy JO, Jackson A, Ken Jee KW, Marks LB, et al. The use and QA of biologically related models for treatment planning: short report of the TG-166 of the therapy physics committee of the AAPM. *Med Phys*. (2012) 39:1386-409. doi: 10.1118/1.3685447

[3] Michalski JM, Gay H, Jackson A, Tucker SL, Deasy JO. Radiation Dose-Volume Effects in Radiation-Induced Rectal Injury. *Int J Radiat Oncol Biol Phys*. (2010) 76:S123-S9. doi: 10.1016/j.ijrobp.2009.03.078

[4] Ong ALK, Knight K, Panettieri V, Dimmock M, Tuan JKL, Tan HQ, et al. Predictive modelling for late rectal and urinary toxicities after prostate radiotherapy using planned and delivered dose. *Front Oncol*. (2022) 12:1084311. doi: 10.3389/fonc.2022.1084311

[5] Emami B, Lyman J, Brown A, Coia L, Goitein M, Munzenrider JE, et al. Tolerance of normal tissue to therapeutic irradiation. *Int J Radiat Oncol Biol Phys*. (1991) 21:109-22. doi: 10.1016/0360-3016(91)90171-y

[6] Panettieri V, Rancati T, Onjukka E, Ebert MA, Joseph DJ, Denham JW, et al. External Validation of a Predictive Model of Urethral Strictures for Prostate Patients Treated With HDR Brachytherapy Boost. *Front Oncol*. (2020) 10:910-20. doi: 10.3389/fonc.2020.00910
